# Spatially-Resolved Study of the Electronic Transport and Resistive Switching in Polycrystalline Bismuth Ferrite

**DOI:** 10.3390/s23010526

**Published:** 2023-01-03

**Authors:** Alexander Abramov, Boris Slautin, Victoria Pryakhina, Vladimir Shur, Andrei Kholkin, Denis Alikin

**Affiliations:** School of Natural Sciences and Mathematics, Ural Federal University, Ekaterinburg 620000, Russia

**Keywords:** scanning probe microscopy, CAFM, defects, leakage current, local switching, polarization reversal

## Abstract

Ferroelectric materials attract much attention for applications in resistive memory devices due to the large current difference between insulating and conductive states and the ability of carefully controlling electronic transport via the polarization set-up. Bismuth ferrite films are of special interest due to the combination of high spontaneous polarization and antiferromagnetism, implying the possibility to provide multiple physical mechanisms for data storage and operations. Macroscopic conductivity measurements are often hampered to unambiguously characterize the electric transport, because of the strong influence of the diverse material microstructure. Here, we studied the electronic transport and resistive switching phenomena in polycrystalline bismuth ferrite using advanced conductive atomic force microscopy (CAFM) at different temperatures and electric fields. The new approach to the CAFM spectroscopy and corresponding data analysis are proposed, which allow deep insight into the material band structure at high lateral resolution. Contrary to many studies via macroscopic methods, postulating electromigration of the oxygen vacancies, we demonstrate resistive switching in bismuth ferrite to be caused by the pure electronic processes of trapping/releasing electrons and injection of the electrons by the scanning probe microscopy tip. The electronic transport was shown to be comprehensively described by the combination of the space charge limited current model, while a Schottky barrier at the interface is less important due to the presence of the built-in subsurface charge.

## 1. Introduction

Memristive properties of the functional oxides are of interest for the development of the new generation of random-access memory (RRAM) [1]. RRAM attracts great attention due to its simple and scalable geometry, the possibility to fabricate multilevel stacking structures, and combine information storage and processing in one chip [1,2]. Fabrication of the RRAM based on ferroelectric thin films is a new milestone for resistive memory devices due to the larger current difference between insulating and conductive states and lower junction activation voltage for resistive switching (which means less heating of the active elements of integrated circuits). Bismuth ferrite (BiFeO_3_, BFO) films are of specific interest due to the combination of high spontaneous polarization and antiferromagnetism [3], implying the possibility to provide both electric and magnetic control. In many reports, BFO demonstrate resistive switching (RS) behavior [4,5,6,7,8,9,10,11,12,13,14,15,16]. However, both proposed mechanisms of the current transport and hysteresis are not apparent and, in all likelihood, depend on the material thickness [4,5], impurity and defect state [6,7,8], and properties of the interface between electrode and material [9,10,11]. In general, RS can have an interfacial origin [12], be stimulated by voltage-induced ionic [1,2,13,14] or electronic processes [15,16], or be a combination of both [7,17,18]. In ferroelectric BFO films, usually, several mechanisms are considered to contribute to RS. RS in ultra-thin epitaxial BFO films is due to the modulation of the potential barrier for electrons tunneling from the electrode into the material by the polarization direction [19,20]. This mechanism is similar to other ferroelectrics and is limited by the thickness of the films of a few nm [21,22], where tunneling can be realized [23,24]. For thicker films, the RS is usually explained by the electromigration of the oxygen vacancies creating conductive “filaments” consisting of trap centers for electrons [25,26,27,28] or modulating the height of the potential barrier at the electrode-sample interface [2,10]. The mobility of the oxygen vacancies in bismuth ferrite is a widely accepted explanation for the RS because of belief of high oxygen vacancies mobility. In addition, the filament formation was revealed by advanced microscopy methods in some oxide materials [13,14], which convinced researchers to propose the analogy over BFO and other materials. One more mechanism is a modification of the potential profile by polarization reversal [5,7,9] or combined polarization reversal/vacancy segregation coupling [29]. A conductivity along the ferroelectric domain walls also provides a track for electronic transport [30,31,32]. As an example, creating/erasing the domain walls was postulated to be responsible for RS in BFO nano-dots [27]. Though a pure electronic trapping/detrapping explanation of RS has been proposed for the explanation of the RS in BFO [6] similar to other materials [33], this hypothesis is not popular. Despite these efforts, the final point in this discussion of RS origin in BFO is not set for now.

The major problem in the macroscopic investigations of charge transport is that the subject becomes more and more difficult, moving away from the ideal semiconductor case. Electrode-material interfaces [17] and elements of microstructure such as domain walls [34], twin and grain boundaries [35,36], dislocations [1]—all of those can impact significantly current transport and RS. Segregation of the structural defects at the domain walls [34], grain boundaries [36], and at the secondary phase locations [37] can modify the type of conductivity [38] and create a net of distributed channels for charge transport. Conductive atomic force microscopy (CAFM) is a useful approach to probe charge dynamics locally in the individual elements of the material microstructure: domain walls [30,31,32], the interior of the material or grain boundaries [36], or even around individual defects [1,39,40]. New technology of fast data acquisition and treatment motivates to further develop CAFM for the studying of disordered and distributed systems [41,42]. Many efforts are taken so far to understand the current transport across the probe-sample interface [43,44], which is important for the correct quantitative interpretation of the local current data. Addressing the issue of the diverse influence of the microstructure in BFO films is important to control the electric transport, which in turn can help to improve the application of BFO in piezoelectric and magnetic sensors, as well as in developing an avenue of the RRAM.

In this work, we introduce a methodology for the local measurements of the current transport using advanced CAFM and reveal the mechanisms of the electronic transport and RS phenomena in polycrystalline bismuth ferrite films. The novel design of the CAFM experiments including simultaneous measurement of the current and piezoresponse, multi-array data analysis of the I–V curves, and supporting measurements by out-of-contact Kelvin probe force microscopy (KPFM), allowed to comprehensively characterize band structure of the material in a completely screened state and after built-in charge uncovering with the application of DC voltage. The Schottky barrier across the probe-material interface was shown to be opened after the built-in charge has been uncovered, and the electronic transport is comprehensively described by the space charge limited current (SCLC) model. Contrary to the many studies by the macroscopic methods, postulating electromigration of the oxygen vacancies, we demonstrate RS to be caused by the pure electronic process of trapping/releasing electrons and the formation of the space charge regions during polarization screening.

## 2. Materials and Methods

Fabrication of BiFeO_3_ thin films was performed by sol-gel route. The solution of dissolved precursors was prepared under stirring and consisted of 0.417 g of bismuth nitrate pentahydrate Bi(NO_3_)_3_·5H_2_O (Sigma-Aldrich 99.9%, St. Louis, MO, USA) and 0.323 g of iron nitrate nonahydrate Fe(NO_3_)_3_·9H_2_O in 1 mL of 2-methoxyethanol (Sigma-Aldrich 99.9%) and 5 mL of glacial acetic acid (Sigma-Aldrich 99.9%). An excess of 7.5 mol% of bismuth nitrate was added to compensate for Bi losses during further annealing steps. After complete homogenization, at 80 °C 3 mL of glacial acetic acid was added to the solution at room temperature after filtering with a microfiber filter paper. Thus, the final concentration was adjusted to 0.16 M. Pt/TiO_2_/SiO_2_/Si(100) substrate for thin film preparation was washed with acetone and 15 layers of BFO film were subsequently deposited by spin coating. After each layer of the film deposition, the substrate was dried at 80 °C for 10 min and then at 125 °C for 40 min. The pyrolysis and crystallization were realized in the air with the two-step annealing procedure: 300 °C for 60 min and 600 °C for 40 min. After annealing, the film was slowly cooled down at a 5 °C/min rate. More details about film preparation and structural characterization can be found in ref. [45].

**Figure 1:**
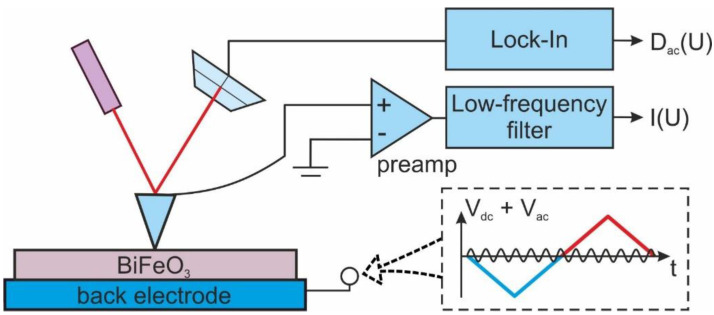
Experimental setup for simultaneous measurements of the current and piezoresponse.

Correlative characterization of the films by scanning probe microscopy methods was done by the scanning probe microscope NTEGRA Aura (NT-MDT, Zelenograd Russia) in the dry-air conditions, processed by the dry nitrogen gas flow through the microscope chamber. The measurements were performed using n-type doped diamond-coated HA-HR/DCP (Scansens, Hamburg, Germany) probes (spring constant 17 N/m). All electrical measurements were performed by the application of the bias voltage to the substrate, while the grounded conductive probe played the role of the top electrode (Figure 1). Thereby, it is implied in all further discussions of polarities, that voltage is applied to the substrate. CAFM measurements were performed using a standard current-voltage amplifier of NTEGRA Aura (STM measurement stage with a built-in current preamplifier). Both internal I–V spectroscopy mode and external program setup based on the NI-6251 data acquisition board (National Instruments Corp., Austin, TX, USA) were used to collect the current from the pre-amplifier. Mapping of the I–V curves was done using internal I–V spectroscopy mode. I–V curves were captured with a 300 ms per point temporal interval. The motion of the probe across the points of the map was done while the probe was withdrawn, which is crucial to reduce the degradation of the conductive probe.

The piezoresponse force microscopy (PFM) imaging was realized by the 3–5 V_rms_ AC voltage at 20 kHz application to the conductive substrate. 2 V_AC_ and 10 V_DC_ voltages were applied through the Trek-677B (Trek, Waterloo, WI, USA) voltage amplifier for PFM spectroscopy. A self-made experimental setup based on the NI-6251 data acquisition board was used for voltage pulses’ application and acquisition of the PFM signals during simultaneous switching and current spectroscopy measurements.

The spatial distribution of the surface potential was visualized in the built-in two-pass closed-loop KPFM mode. 0.1 V_AC_ voltage was applied to the conductive substrate during the second pass at a 1 nm lift distance.

## 3. Results and Discussion

### 3.1. Piezoresponse and Conductivity Distribution across the Grains

**Figure 2:**
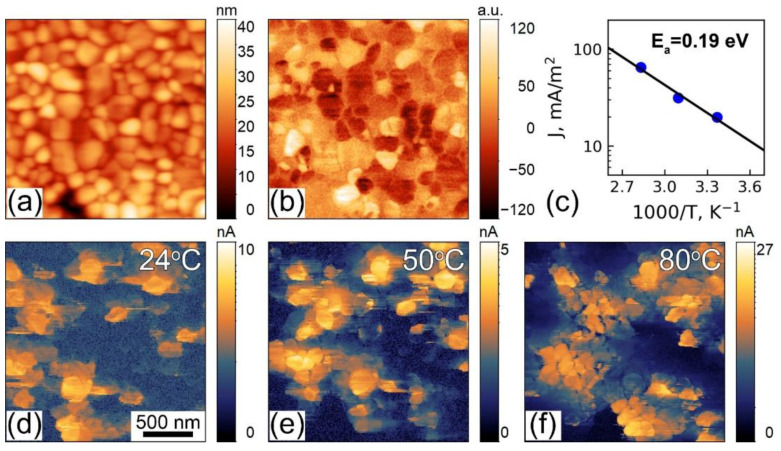
BFO thin film: (**a**) Topography, (**b**) out-of-plane PFM, (**c**) dependence of C-AFM current density (J) on reverse temperature, (**d**–**f**) CAFM in BFO thin film in dependence on temperature: (**d**) RT, (**e**) 50 °C, (**f**) 80 °C.

First, we measured out-of-plane piezoresponse and current distribution across the grains of the films using correlative PFM and CAFM measurements, respectively (Figure 2). PFM revealed randomly distributed piezoresponse in the grains (bright and dark contrasts in Figure 2b). At the same time, CAFM measurements at 10 V DC voltage show the appearance of conductive regions (low resistance states, LRS) usually spatially correlated with the position of the grains (Figure 2d), i.e., some of the grains were conductive and some remained insulating (high resistance state, HRS). The magnitude of the current usually ranged from hundreds of pA to a few nA at room temperature. Increasing the temperature leads to the growth of the film area with enhanced conductivity (Figure 2d–f), while the distribution of the piezoresponse remained unchanged (not shown here). The current density was calculated by dividing the integral current across the scan by the total area of conductive regions and plotted against reciprocal temperature. The obtained dependence was fitted by the Arrhenius relation (Figure 2c). The extracted activation energy is about 0.2 eV, which is in the range of the activation energy for small polaron hopping in perovskite materials [46]. Similar values of activation energies were observed in BFO polycrystalline films [36,47,48] and ceramics, processed in an oxygen-rich atmosphere [49]. RS behavior has been revealed by the application of the triangular voltage pulses with a maximum of 10 V to a scanning probe microscopy (SPM) probe and further scanning in CAFM mode at a lower applied DC voltage (Supplementary Materials S1, Figure S1). After the application of the voltage pulse during 300 ms, the region of the grain became conductive (Figure S1). The result of the experiment was similar to that reported in ref. [26], while localized current channels, reported for TiO_2_ [13] and SrTiO_3_ [14], were not observed. Importantly, the film conserved conductive states for days. To get a further deeper insight into the RS mechanisms, current-voltage (I–V) dependencies were acquired in a spatially-resolved manner.

### 3.2. Clustering of I–V Curves

**Figure 3:**
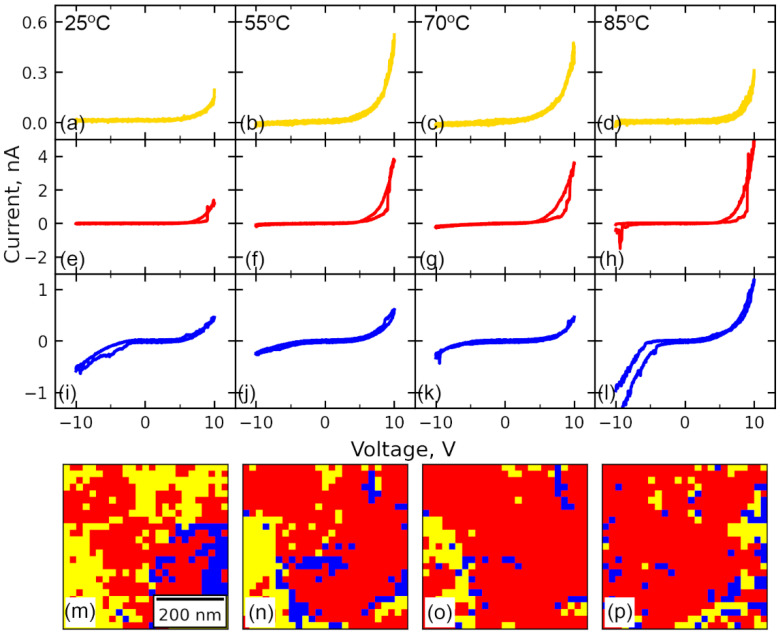
Typical IV dependencies: (**a**–**d**) unipolar, (**e**–**h**) unipolar with RS, (**i**–**l**) bipolar, and (**m**–**p**) their distributions at various temperatures: (**a**,**e**,**i**,**m**) 25 °C, (**b**,**f**,**j**,**n**) 55 °C, (**c**,**g**,**k**,**o**) 70 °C, (**d**,**h**,**l**,**p**) 85 °C.

I–V dependencies were collected across the array of the points in several grains of the film to comprehensively characterize the heterogeneity of the conductivity. The measurements were done in the same regions at various temperatures. The shape of the hysteresis was different in the different regions at the surface (Figure 3). I–V dependencies can be conventionally separated into three specific groups: (1) highly unipolar curves with characteristic rectifier behavior [17], where current is registered only under positive applied voltage (yellow curves in Figure 3a–d), (2) hysteretic unipolar curves with a current “jump”, characteristic for the RS (red curves in Figure 3e–h), (3) symmetric weak-hysteretic curves (blue curves in Figure 3i–l). The hysteresis occurred mostly under the positive polarity of the voltage, while small hysteresis can be observed for the negative polarity of the voltage in the points, where current-voltage characteristics were symmetric or at elevated temperature (Figure 3h). The spatial distribution of the curves of the different types across the area of the scanning is presented in Figure 3m–p. The criteria of the rectifying ratio higher than 100 were chosen to separate yellow curves, while the curves with RS (opening of the hysteresis under the positive voltage) were separated into the group specified by the red color.

**Figure 4:**
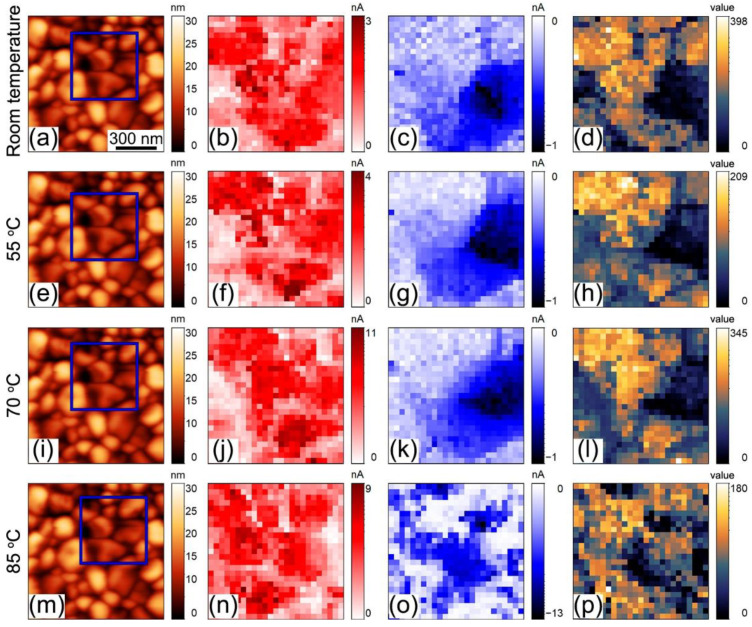
IV dependencies mapping at various temperatures: (**a**–**d**) 25 °C, (**e**–**h**) 55 °C, (**i**–**l**) 70 °C, (**m**–**p**) 85 °C. (**a**,**e**,**i**,**m**) Topography; current in log-scale (**b**,**f**,**j**,**n**) at U = 10 V and (**c**,**g**,**k**,**o**) at U = −10 V; (**d**,**h**,**l**,**p**) coefficient of asymmetry: current amplitude at 10 V divided on current amplitude at −10 V. Thermal drift is present in (**m**–**p**). Blue squares in (**a**,**e**,**i**,**m**) indicate the region at the scan, where I–V curve mapping was done in the respective row.

Further analysis of the material heterogeneity was done by plotting the parameters of the obtained I–V curves. The distributions of the current value at −10 V and +10 V applied DC voltage and ratio between these two values, indicating asymmetry of the current or “rectification ratio”, are plotted in Figure 4. The current distribution at U = 10 V at room temperature (Figure 4b), partially correlates with the topography and differs in the different grains. Some of the grains were more conductive than others and the last cannot be attributed to the topography or piezoresponse features (Supplementary Materials S2, Figure S2). At U = −10 V the current is absent almost everywhere across the map, except the region of almost symmetric I–V curves (dark-blue region in Figure 4c,g,k). The area occupied by the non-hysteretic type of curves decreases with the temperature being exchanged by the curves with hysteric behavior (Supplementary Materials S3, Figure S3a). At 70 °C current hysteresis was observed almost over the whole map.

**Figure 5:**
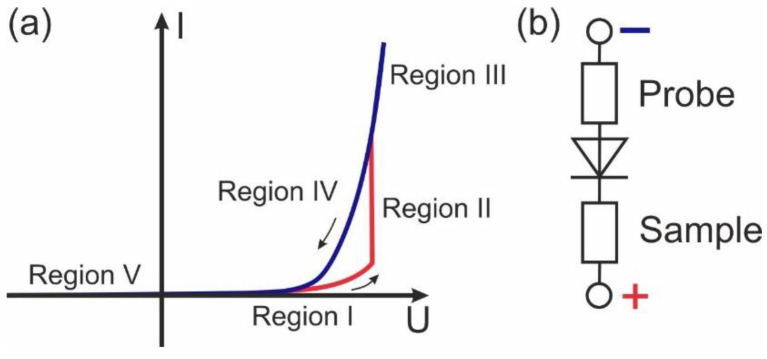
(**a**) Schematics of the hysteretic I–V dependencies with RS obtained in BFO films. Arrows indicate the counterclockwise (CC) direction of the bypass. (**b**) Lumped-based scheme of the probe, sample, and Schottky-barrier in the CAFM experiment.

The hysteresis of the current in the red curves of Figure 3m–p can be divided into five regions (Figure 5a). In Region I—typically higher 3–4 V (HRS), a small current is registered. In Region II, under the application of the positive voltage larger 5–6 V rapid increase of the current (“jump”) is observed, manifesting a transition to LRS. In region III, both forward and backward curves of the current are coincident. In Region IV, the current continues to decrease along the trajectory of Region III, In the backward pass (Region IV) the current goes by a different route than in Regions I and II, which forms a hysteresis of the resistivity. Keeping in mind the temporal resolution of the measurements, RS happens at the timescale of below 10 μs, which manifests the pure electronic origin of the process. Finally, in Region V current is absent or small. At elevated temperatures, the current flow was observed for the voltage of both polarities in some spatial points.

### 3.3. Band Structure in Completely Screened and Unscreened BFO

Typically, rectifying behavior indicates a Schottky contact between the probe and material [17]. Contrary to the case of macroscopic measurements, where both interfaces take a part in the current flow [9], CAFM inspects the current in the vicinity of one chosen interface, and thereby only one Schottky barrier contributes to the current flow (Figure 5b), which is due to orders of difference between tip contact area and an area of the bottom electrode. To verify the concept of the Schottky barrier at the interface, the band structure of the probe-material interface should be analyzed.

BFO films can exhibit both p-type and n-type semiconductor properties, which depend on the off-stoichiometry [49,50,51]. The measurements by X-ray photoelectron spectroscopy (XPS) didn’t reveal a valuable amount of oxygen vacancies in BFO film (Supplementary Materials S4, Figures S4 and S5). At the same time, the segregation of bismuth at the surface can indicate the presence of the bismuth vacancies underneath, which, however, were not seen to increase the amount of Fe^4+^ ions. Thus, the films can be proposed either to be p-conductive due to the small bismuth loss or exhibit an *n = p* intrinsic state, where both electrons and holes are responsible for the current transport. In further discussion, we assume BFO to be a p-type semiconductor but comment on the influence of chemical heterogeneity on the semiconductor properties of the material.

To get an insight into conduction mechanisms, contact potential difference was probed. For that, KPFM measurements were performed at freshly cleaved pyrolytic graphite (HOPG) and epitaxial (100) platinum film, washed in acetone before measurements, and BFO. The results of the measurements are summarized in Table 1. Accepting the work function of HOPG to be 4.6 ± 0.1 eV [52], the work function of the diamond-coated tip was evaluated to be 4.85 ± 0.1 eV, and the work function of the BFO was around 4.78 ± 0.1 eV. Importantly, the measurements were done out-of-contact, before I–V measurements using CAFM, and thereby they represent undisturbed inspection of the contact potential difference in a fully screened sample. In ferroelectrics, polarization is usually compensated by the charged defects and adsorbates at the surface [53], which realizes the screening of the depolarization field [54]. The polarization can be expected to be partially unscreened under the application of DC voltage to the probe [54].

**Figure 6:**
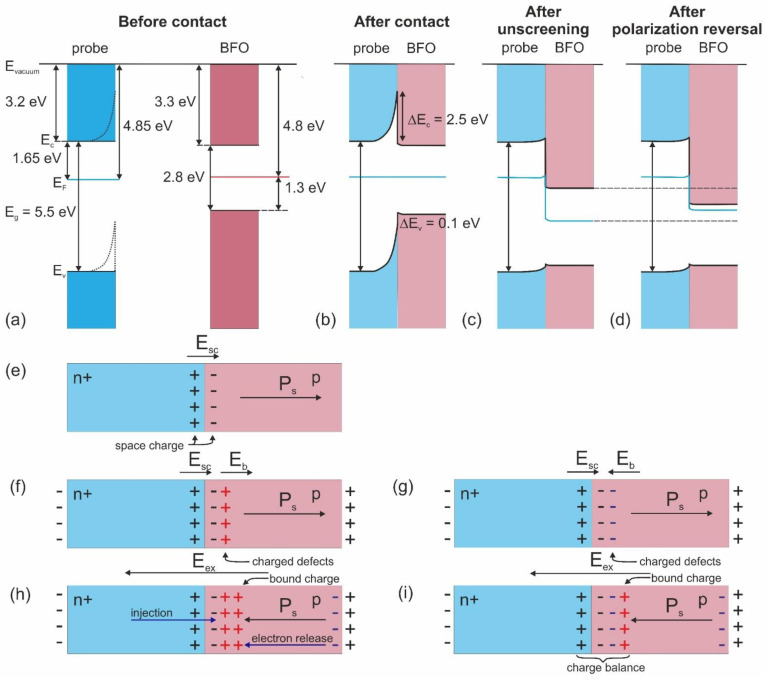
(**a**–**d**) Band diagrams of the doped n+-diamond-coated probe and p-type BFO: (**a**) before contact, (**b**) after contact, (**c**) after the unscreaning, (**d**) after polarization reversal. (**e**–**i**) Distribution of the charges: (**e**) in fully screened BFO, (**f**,**g**) after unscreening of (**f**) positive and (**g**) negative built-in charge, (**h**,**i**) after polarization reversal in the case of (**h**) positive and (**i**) negative built-in charge.

Based on KPFM measurements and literature data for the electron affinity and energy band gap [55,56], band diagrams for highly-doped n+ diamond probe coating and BFO can be reconstructed (Figure 6). It should be mentioned that this consideration is simplified, because of neglecting contamination at the surface, surface stress, and other factors, which can modify the surface states of the semiconductor. The position of the Fermi level is only slightly different for both materials (Figure 6a). They would form an Ohmic contact if the probe doesn’t have a band bending near the surface [55]. Such a bending (even being reduced as a result of contact with p-type BFO) produces a barrier for electrons preventing Ohmic current flow. The Schottky barrier exists also for holes, which cannot penetrate from BFO to the metal probe due to the energy difference between valence bands (Figure 6b).

**Figure 7:**
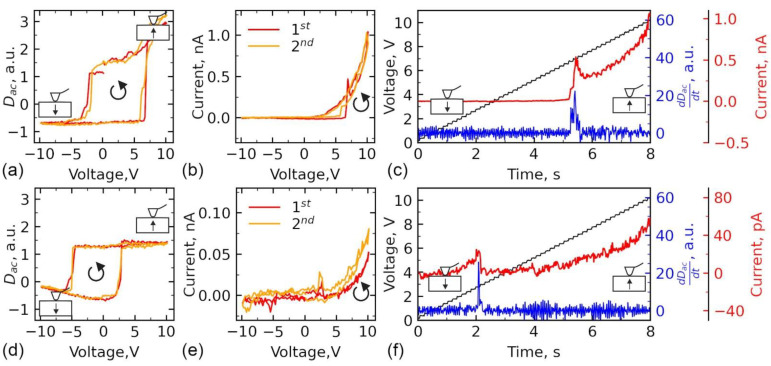
Simultaneous measurements of the piezoresponse (D_ac_) and current hysteresis for (**a**–**c**) positive and (**d**–**f**) negative built-in surface charge. (**a**,**d**) Piezoresponse and (**b**,**e**) current hysteresis loops. (**c**,**f**) Temporal dependencies of the dD_ac_/dt—piezoresponse derivative (blue) and current (red). The bypass of the loop is counterclockwise (indicated by the circled arrow). Before the measurements of the hysteresis loop, negative 10 V, 1 s duration triangular-shape pre-pulse was applied to the substrate to set up the direction of the polarization equally upward.

The band diagram in Figure 6b is valid only for the completely screened ferroelectric surface. Polarization reversal induces interfacial charge and attracts carriers for screening, which can change the position of the Fermi level in semiconductor ferroelectrics [9]. To verify the contribution of the polarization reversal and screening, the experiment with simultaneous registration of the current and piezoresponse were performed. Step-like triangular DC voltage pulses overlapped with AC voltage were applied to the substrate, while current and piezoresponse signals were measured (Figure 1). The piezoresponse signal was plotted as is, while the current signal was filtered from a high-frequency AC component utilizing a low-pass filter. The grains with initially upward and downward-directed polarization were chosen for the experiments by monitoring the piezoresponse signal. The positive triangular pre-pulse was applied to the substrate in all grains before measurements of the I–V curves to orient polarization equally upward. Typical piezoresponse, D_ac_ (Figure 7a,d), and current (Figure 7b,e) loops measured in the points with initially different directions of polarization are presented in Figure 7. Temporal dependencies of the piezoresponse derivative dD_ac_/dt and current signal, during the application of the positive part of the voltage spectroscopy waveform followed by the negative part (Figure 1), are plotted in Figure 7c,f. Despite the equal direction of polarization during switching after pre-pulse, the results differ significantly for the grains with the polarization initially oriented upward or downward. The apparent difference of the bias field, i.e., the shift of the hysteresis along the axis of voltages, can be noticed (Figure 7b,e). The origin of the bias field can be a built-in charge localized under the probe [54]. As distinguished from the results in ref. [54], DC voltage is applied to the substrate, which assumes a correlation between the sign of the bias field and the sign of the built-in charge (correlation instead of anti-correlation in ref. [54]). Similar to the case of bulk ceramic BFO, built-in charge can be attributed to the segregation of the charged defects or free carriers taking a part in the polarization screening. Indeed, domain states with initially upward-directed polarization have a negative built-in charge, and domain states with initially downward-directed polarization have a positive built-in charge. It should be noted that built-in charge doesn’t spread out during the time of pre-pulse and hysteresis loop measurements, which is seen in the conservation of the bias voltage in the loop during cycling.

An apparent correlation between the sign of the bias field in the loop and RS is seen (Figure 7a,b,d,e). In the case of the positive bias field, RS happens (Figure 7c), while it is absent in the case of the negative bias field (Figure 7f), as well as the total level of the current is much lower in this case. The analysis of dD_ac_/dt and current temporal dependencies (Figure 7c,f) allows to compare events of the polarization reversal and RS. The small increase of the current just in the moment of polarization switching is likely a switching current [57] or a current flowing along the conductive domain walls during switching [58]. While the large jump in the current is coincident with the peak of dD_ac_/dt, i.e., nucleation bias of polarization reversal (corresponding to the threshold field of the polarization reversal).

To explain the polarization reversal contribution to the change of band structure, the influence of interfacial charge on band bending should be analyzed. As we discussed above, the contact between n+-diamond and p-type BFO is a Schottky-type for completely screened ferroelectric, i.e., when it can be considered simply as a normal semiconductor (Figure 6b,e). Increasing DC voltage at the probe in contact with the surface leads, first, to uncovering of the subsurface built-in charge by removal of the surface contamination (Figure 6f,g) and then to polarization reversal (Figure 6h,i). In the beginning, we consider the case of the positive built-in charge at the surface (Figure 6f,h and Figure 7a–c). The bias voltage of the piezoresponse loop is +2.5 V (Figure 7a), while the nucleation bias is 4.6 V (Figure 7c). When applying an electric field, the built-in charge acts against the direction of applied voltage and reduces an electric field created by the SPM probe in the material (Figure 6f). Taking in mind that the height of the Schottky barrier is 2.5 eV and the bias voltage is 2.5 V, a positive voltage higher than 5 V should be applied to overcome the Schottky barrier height by moving Fermi energy in BFO down and establishing the Ohmic regime for current flow (Figure 6c). That is coincident with the “cut-off” voltage (the voltage before which the current is absent) in I–V dependence in Figure 6b, which is also approximately equal to nucleation bias in this spatial point.

In the case of a negative built-in charge, the bias voltage is −1 V (Figure 7d) and the nucleation bias is 3.8 V (Figure 7f). Thus, the bias field, on the contrary, renders to lower the Fermi level together with the external field, which leads to overcoming the potential barrier below 1.5 V applied external voltage, which is again coincident with the “cut-off” voltage for the current flow (Figure 7e) and close to the nucleation bias of about 2 V.

Application of the negative voltage to the BFO film stimulates the processes, which are opposite to those discussed above. It should increase the height of the Schottky barrier for the cases of both polarities of the bias field, which is responsible for the rectifying behavior observed in the experiment (Figure 3e,i).

Thus, the Schottky barrier is opened just in the moment or before polarization reversal. However, a large difference of I–V curves for the case of the positive and negative bias field in ferroelectric implies that not a Schottky barrier is responsible for RS, because, in the same conditions of open Schottky barrier, RS occurs only for the case with the positive built-in charge. Additionally, a significant difference between the “cut-off” voltage and threshold field of RS exists (Figure S3b), which contradicts the model of Schottky barrier opening during polarization reversal. From the point of the impact to the band structure, the appearance of the polarization bound charge leads to the lowering of the conduction band with respect to Fermi level (Figure 6d), i.e., band bending in the local region of the material. BFO is very close to intrinsic semiconductor, and an effective increase of the Fermi level is equivalent to the change of the conductivity type from the p-type to the n-type. This change influences the mechanism of electron transport, which will be discussed in the following paragraph.

### 3.4. The Mechanisms of Electron Transport and Resistive Switching

**Figure 8:**
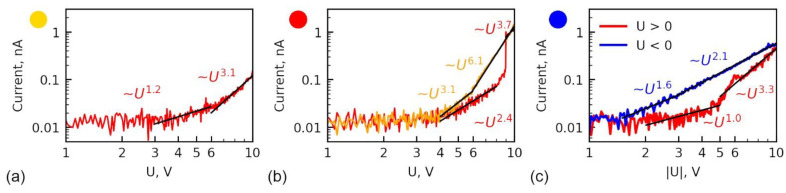
Analysis of (**a**) non-hysteretic I–V dependencies from Figure 3a, (**b**) hysteresis I–V dependencies with RS from Figure 3e, (**c**) symmetric I–V dependencies from Figure 3i. The red and orange colors in (**b**) respect to the voltage rise and fall. The color circle indicates the type of the curve according to maps in Figure 3m–p.

To clarify the mechanisms of the current transport under the voltages of the different polarities, we further analyze all three types of I–V dependencies at room temperature: asymmetric non-hysteretic (Figure 3a), asymmetric hysteretic (Figure 3e) and symmetric (Figure 3i), Three curves from Figure 3a,e,i are presented in Figure 8 in log-log scale.

The asymmetric current at the red curve in Figure 8a is related to the negative built-in surface charge. The rectifying behavior assumes is caused by the formation of a Schottky barrier. As was explained in the previous section, the Schottky barrier exists only for negative applied voltages, while at low positive voltages (below 1.5–2 V), i.e., built-in negative charge removes the barrier (Figure 6c). To double-check that the mechanism of the current transport under positive voltage is not limited by the Schottky barrier, I–V curves were fitted with the equations of a current through the Schottky barrier:

(1) $$I=AT^{2}\exp-\left(\frac{\Phi }{k_{b}T}-\frac{1}{k_{b}T}{\left(\frac{q^{3}V}{4\pi \varepsilon \varepsilon_{0}d}\right)}^{\frac{1}{2}}\right),$$

where A—Richardson constant, $k_{b}$—Boltzmann constant, *T*—temperature, q—elementary charge, V—applied voltage, Φ–height of Schottky-barrier, ε—dielectric constant of the film, d—film thickness. Fitting yields the unrealistic value of dielectric constant below 7 (Supplementary Materials S5, Figure S6a), which is expected to be around 30, according to molecular dynamics simulations [59] and for sol-gel films is usually higher due to the contribution from leakage current [36].

To inspect the mechanism of the current flow, the fitting of the I–V curve by the linear function in the log-log scale was performed. Its slope indicates the power degree of the polynomial fitting curve. In the positive branch of current (red curve in Figure 8c), in the range below 5 V, the slope is around 1, which assumes Ohmic current. At higher DC voltages, the slope becomes higher, ≈3, which assumes a transition to SCLC regime of the current flow, caused by the charge injection from the SPM probe [60]. It can be formally described by the following equation:

*I* = *aV^m^*, (2)

where *a* is the constant, which depends on the parameters of the band diagram and the defect state of the material (density of the trapping centers for electrons). Power degree *m* characterizes the mechanism of the current flow: *m* = 2 for the insulator without traps (Mott-Gurney regime) and *m* is higher than 2 in the case of deep trap existence (modified Mott-Gurney regime) [60,61]. Importantly, the degree *m* can be indicative of the sort of traps and their occupancy [60]. The higher the power degree *m*, the more energy levels contribute to electronic transport. In Rose-Lambert theory for defect-free semiconductors, the power *m* is limited by 3–4, which is determined by the ratio of the temperature of the measurement and temperature of the trap appearance (sintering temperature) [60,61]. Including into consideration the contributions from the dopants (defects) and Pool-Frenkel effect renders a much wider possible variation of *m*, which is determined by the density of the defect and their energy in the band gap [62]. Equation (2) is thus written in general form, because it has an analytical solution only for the simplified case of the Rose-Lambert theory [60,61], while the analytical solution is absent for the model including dopants and Pool-Frenkel effect [62]. Nevertheless, power degree *m* can be used for the qualitative interpretation of the current transport and respective material defect state according to the rule “the lower degree of *m*, the traps are more filled”.

Further, the fitting by Equation (2) was performed for the analysis of RS in the hysteretic curve from Figure 8b. Similar to the above discussion of the non-hysteretic curve, Equation (1) is irrelevant to fit any region of the curve with the reasonable output parameter (Supplementary Materials S5, Figure S6b), while SCLC fits well all three regions of the curves (Figure 8b). In region I before RS (HRS), *m* has a value close to 2, i.e., trap-filled state, while after RS in region III (LRS) the value of *m* increases almost twice to m ≈ 4, which indicates a state with a large number of free traps [62]. Such a change assumes a release of the electrons from the deep traps to the conduction band as a mechanism of the current “jump” and subsequent refilling of the traps by the electrons injected from the SPM tip. In the backward pass of the current—region IV (LRS), *m* remains high, which again signifies that the traps are freer of electrons in comparison to the forward pass.

As we discussed earlier, the threshold voltage of the RS is coincident with the threshold voltage of the polarization reversal (Figure 7). Keeping in mind an analysis of the band structure in the previous section, the following scenario can be suggested to explain the effect of RS in BFO. Polarization reversal leads to an instant lowering of the conduction band bottom, which reduces the energy necessary for the electrons in the deep traps to be released to the conduction band. Appearing positive polarization bound charge needs carriers to screen the depolarization field of newly created domains. As a result, electrons are released from the deep traps to the conduction band providing carriers to screen the polarization charge, while their position is refilled by the electrons injected from SPM tip.

**Figure 9:**
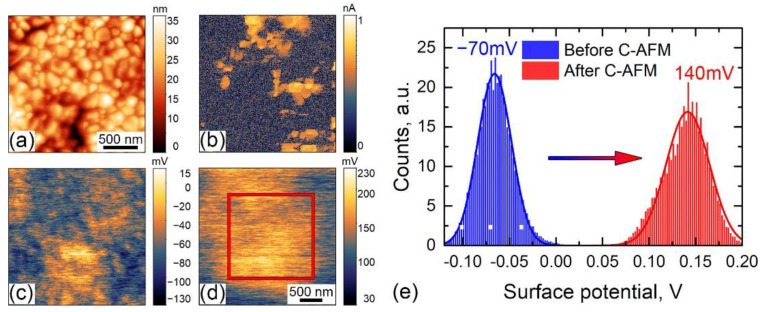
(**a**) Topography, (**b**) current distribution after RS, and surface potential distribution in BFO film (**c**) before and (**d**) after RS, (**e**) histograms of surface potentials. The red square—is a scanning region at (**a**–**c**).

This hypothesis was confirmed by monitoring surface potential after RS experiments (Figure 9). At first, KPFM scanning was performed in the region of interest to probe undisturbed contact potential difference (Figure 9a,c). Further, the same region was scanned by CAFM at +10 V DC, which induces RS in several regions at the scan (Figure 9b). Finally, KPFM scanning was performed again (Figure 9d). The surface potential changes from −70 mV in a completely screened state to 140 mV, which corresponds to the negative screening charge, i.e., electrons. Partly, the change of the surface potential can be explained by the injection of the electrons from the SPM tip to the conduction band, but current “jump” in I–V curves occurs for a time span of few μs, while typically charge injection from the SPM probe is much slower—at the level of ms [63]. Again, RS cannot be explained simply by the opening of the Schottky barrier, because, in this case, we should see a surface potential with negative polarity respective to the polarization-bound charge. In our case, the surface potential is positive, and the opened state of the Schottky barrier is due to the lowering of the conduction band. Further charge injection also rises the Fermi level, which enhances the electron conductivity of the BFO.

An apparent similarity between the enhanced conductivity during RS and conductivity along the domain walls should be mentioned. For the case of BFO, conductivity after RS has the same activation energy ~0.2 eV as the conductivity along the interfaces in thin BFO films: domain walls and phase boundaries [64], which assume the same origin. In both cases, the enhanced conductivity is a result of the band bending under the interfacial bound charge and represents a screening charge flow. A release of the electrons to the conduction band can happen not only in the vicinity of the surface, but the process can further spread into a material bulk with a continuous injection of the electrons from the probe stimulating transition of the larger volume of the material to LRS. The mechanism of this LRS dissimilation was previously described for BTO films [65].

The difference between hysteretic and non-hysteretic unipolar curves in Figure 9a,b, respectively (yellow and red regions in Figure 3m–p) is due to the sign of built-in charge: positive for the red region and negative for the yellow region. The variation of the built-in charge can be associated with the segregated charged defects of the different polarities, such as oxygen vacancies and bismuth vacancies [54]. After polarization reversal in the case of the positive built-in charge (red curves), the polarization is not compensated by any carrier. At the same time, most of the trap centers are occupied by electrons or become occupied under the electron injection to the deep traps (for example, the same oxygen vacancies). It further triggers the release of electrons from the deep traps to screen the polarization. In the yellow regions in Figure 3m–p, the situation is different, because the bound charge after polarization reversal is by part compensated by the negative bound charge and can be screened by the electrons injected from the SPM probe to the free deep traps and the conduction band. Thus, charge balance can be achieved without electron release (Figure 6i).

Before analysis of the symmetric curves from Figure 3i, we should emphasize the influence of the temperature on the current. On the one hand, the threshold voltage of RS (voltage of current “jump”) significantly decreases with temperature (Supplementary Materials S3, Figure S3b). On the other hand, the area covered with symmetric and asymmetric non-hysteretic curves decreases in favor of symmetric hysteretic curves (Supplementary Materials S3, Figure S3a). At elevated temperatures, RS occurs almost in the whole area of the film, and electron transport properties become more homogeneously distributed across the surface (Figure 4m–p). This behavior can be explained by the rise of the Fermi level closer to the intrinsic *n = p* state, which is a general trend for semiconductors, and the total enhancement of the electron concentration due to the thermalization of the carriers. The excess of the carriers leads to the faster occupation of the electron traps and thus faster occurrence of the electron release. Importantly, at an elevated temperature, the negative branch is non-blocking anymore, which is due to the removal of the Schottky barrier for electrons by the change of the Fermi level position in relation to the conductance band.

Returning to symmetric curves from Figure 3i, an absence of the Schottky barrier can be noticed and thus propose their origin to be similar to the case of elevated temperature. The power degree *m* for the positive branch of the current is quite similar to non-hysteretic symmetric curves (Figure 9c). In the range below 5 V, the slope is around 1, which assumes the Ohmic current. At larger DC voltage, the slope becomes larger, 3.3, which is the SCLC regime. In the negative voltages, SCLC has *m* ~ 2 in the whole range of voltages with a small deviation from this law at low voltage. *m* ~ 2 indicates a high degree of trap occupation, which is unable to be disturbed by the applied external voltage. An absence of the Schottky barrier can be attributed to a higher position of the Fermi level in BFO in this region, which can be caused, for example, by the elevated concentration of positively charged defects, such as oxygen vacancies (much larger than in red areas in Figure 3m–p), releasing extra electrons to the conduction band. It locally changes the conductivity type in BFO to n-type and removes a potential barrier between the probe and material.

**Figure 10:**
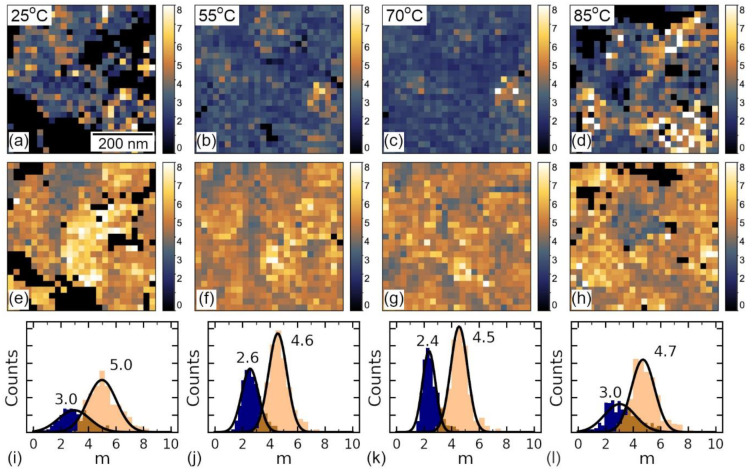
The distribution of power degree m extracted by the fitting of I–V curves from Figure 3 by Equation (2) in (**a**–**d**) forward and (**e**,**f**) backward direction of the positive current branch. Black dots denote the region, where the current is small and fit doesn’t coverage. (**i**–**l**) Respective histograms extracted from (**a**–**d**) are plotted in blue and from (**e**–**h**)–in the beige color.

Thus, in all three cases, SCLC model describes well the mechanism of the current transport, while the main difference of the regions is likely in the trap occupancy, which can be caused by the non-uniform distribution of the charged defects [54]. The density of the trapping centers, i.e., in-gap charged defects providing carriers to the conduction zone, can be estimated by the evaluation of the power degree *m*, the parameter of the fitting in Equation (2). In Figure 10a–d, the spatial distribution of the parameter *m* across the region from Figure 3m–p was plotted, which was achieved by the fitting of the forward (Figure 10a–h) backward pass of the I–V curve positive branch by Equation (2). Respective histograms of the distribution of the power degree *m* degree are plotted in Figure 10i–l. The comparison of the histograms of power degree before and after RS directly shows an almost double increase of the power degree from ≈2 to ≈4, which indicates a change of the material band structure from trap-occupied, before RS, to the state with free traps, after RS, as a result of the electron release to the conduction band.

In contrast to the curves from the red area from Figure 3m–p, the power degree *m* in the yellow and blue areas does not show an apparent change after I–V measurements (Figure 10), which shows a similar trap occupation before and after voltage application in these regions. After electron release, traps in yellow and red regions have a similar distribution, which shows an apparent similarity of these states, despite the sort of charged defects: initial built-in negative charge or bound electrons screening the newly created state of the polarization. In blue regions, the power degree *m* is around 4–5, but the behavior of the curves is non-hysteretic, and the Schottky barrier is likely absent. This region can be thought to be more filled with oxygen vacancies giving electrons to the conduction band, which results in a higher position of Fermi level and n-type of conductivity in the initial state. However, in-depth insight into the local defect state is necessary due to unknown sign of the built-in charge in this region. At elevated temperatures, the distribution of power degree *m* becomes more homogeneous, which demonstrates faster redistribution of the carriers across the traps. In addition, the value of the current “jump” during RS in Figure 3m–p increases with increasing temperature (Supplementary Materials S6, Figure S7), which is also in good agreement with the model of the trap release [65].

Finally, we would like to expand the conclusion about the mechanism of the RS into a macroscopic scale. Recently, the combined mechanism of opening the Schottky barrier and further trapping of the electrons by polarization was proposed for BFO films [7,9]. On the basis, of the current findings, we argue that the Schottky barrier is unlikely to describe the observed behavior. Similar to our analysis of the local I–V curves, the macroscopic curve from ref. [9] was analyzed (Supplementary Materials S7, Figure S8). The mechanism of the current transport is postulated to be the same as in our research, i.e., the release of the electrons from the deep traps triggered by polarization reversal and aimed to screen the polarization bound charge.

## 4. Conclusions

By application of advanced CAFM, significant heterogeneity of the electronic transport in polycrystalline BFO has been revealed, which renders the spatial distribution of the Fermi level across the film surface. The in-depth analysis of local I–V curves through the inspection of space charge limited current parameters and monitoring the bias field of the local piezoelectric hysteresis loops allows us to evaluate the spatial distribution of the trapping centers and connect it with the localization of the charged defects. RS in BFO was shown to be completely determined by the electronic processes triggered by the polarization reversal and screening without using the concept of mobile oxygen vacancies. The hysteresis of the conductivity can be explained by the bending of the conduction band under the action of polarization-bound charge and the subsequent release of the electrons from the deep traps, which screen the polarization. RS switching was shown to be largely determined by the localization of the charged defects. The avalanche-like increase of the electronic current occurs mostly in the region with localized positive built-in charge while it is absent for negative built-in charge, which can be explained by the difference in the conditions of the polarization screening. The suggested mechanism of RS can be expanded to the case of the macroscopic BFO capacitors, which were shown to behave similarly. The proposed CAFM-based method to study local transport properties is not limited to BFO. The inspection of the electron trap density is important for the spatially-resolved study of electronic transport in many modern semiconductor materials. While a deeper understanding of the mechanisms of the current transport and origins of the heterogeneity in BFO films is important to further adapt them for piezoelectric and magnetoelectric applications.

## Figures and Tables

**Table 1:** The results of the surface potential measurements for three different samples and its recalculation to work function.

	HOPG	(100) Pt	BFO
Surface potential	−250 mV	−50 mV	−70 mV
Work function	4.6 ± 0.1 eV from ref. [52]	4.8 ± 0.1 eV	4.78 ± 0.1 eV

## Data Availability

The initial data presented in this study are available on request from the corresponding author. The data are not publicly available due to large data size.

## Supplementary Materials

The supporting information can be downloaded at: https://www.mdpi.com/article/10.3390/s23010526/s1, References [66,67,68,69,70] are cited in the supplementary materials. Figure S1: Topography, CAFM before and after the application of the triangular pulse; Figure S2: Topography, piezoresponse, and CAFM of the region from Figure 2 in the main text of the manuscript; Figure S3: Temperature dependencies of the area with the I–V curves of the different type with a color in correspondence to the Figure 2 from the main text of the paper. Temperatures dependencies of the RS threshold voltage and “cut-off” voltage of the registered current; Figure S4: Comparison of the measured Fe 2p_3/2_ XPS spectra of the as-loaded and etched by Ar ion beam BFO films with the reference literature data for the Fe_2_O_3_ powder, BFO single crystal, and modeled PFN ceramics spectra and different literature reference data for the materials containing Fe^4+^ ions at the surface: before and after the shift of the spectra at 1.1 eV to higher binding energies. Fe^2+^ band at ~708 eV is indicated; Table S1: Atomic concentrations of the elements calculated from XPS spectra; Figure S5: Deconvolution of Fe 2p_3/2_ band to Fe^3+^ and Fe^4+^ multiplet components for as-loaded and etched by Ar ion beam BFO films; Figure S6: Fitting of the positive branch of the non-hysteretic and hysteretic I–V curves from Figure 2e,i by the Equation (1) from the main text of the manuscript; Figure S7: The maps of current “jump” during resistive switching at 25 °C, 55 °C, 70 °C and 85 °C extracted from I–V curves; Figure S8: Analysis of the macroscopic I–V curve obtained for the epitaxial BFO film (Au/BFO (150 nm)/SrRuO_3_ (20 nm)/SrTiO_3_ (001) grown from a Bi_1.05_FeO_3_ target) utilizing SCLC and trap release/refill model.
