# Supplementary material for: Spatially-Resolved Study of the Electronic Transport and Resistive Switching in Polycrystalline Bismuth Ferrite
Source: Sensors (Basel). 2023 Jan 3;23(1):526. doi: 10.3390/s23010526 (PMC9823478; doi:10.3390/s23010526)
Supplement: Supplementary file 1 [file sensors-23-00526-s001.zip › sensors-2123687-supplementary.pdf]

Supplementary Materials

Spatially-resolved study of the electronic transport and resistive switching in polycrystalline bismuth ferrite

Alexander Abramov, Boris Slautin, Victoria Pryakhina, Vladimir Shur, Andrei Kholkin, Denis Alikin*

School of Natural Sciences and Mathematics, Ural Federal University, 620000 Ekaterinburg, Russia;
alexander.abramov@urfu.ru; boris.slautin@urfu.ru; viktoria.pryahina@urfu.ru; vladimir.shur@urfu.ru; kholkin@urfu.ru;
* Correspondence: denis.alikin@urfu.ru;

S1. Resistive switching by the application of the triangular pulses

The region of $BiFeO_3$(BFO) film was scanned by conductive atomic force microscopy (CAFM) with 5V applied DC voltage, which is below the threshold voltage of RS. The conductivity was absent in the region of interest before the voltage pulse application (Figure S1b). 300ms triangular voltage pulse with a maximum 10V DC positive voltage was applied to the center of the grain, and then the region of the film was scanned again by CAFM with 5V applied DC voltage (Figure S1d). CAFM image revealed a clear transition of the film from a high resistance state to a low resistance state.

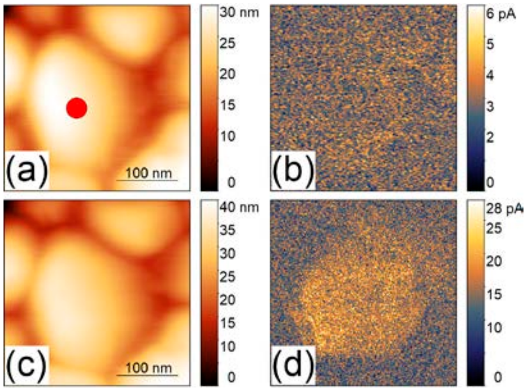

Figure S1. (a), (c) Topography, (b), (d) CAFM; (a), (b) before and (c), (d) after the application of the triangular pulse. The red dot is the point of voltage pulse application.

S2.Topography, piezoresponse, and CAFM of the region from Figure 3

The PFM measurements were performed before the I-V map measurements at room temperature presented in Figure 2m in the main text of the manuscript. 3V AC modulating voltage was used in PFM measurements (Figure S2b). The current distribution was investigated with an applied 10 V DC voltage after the capture of the I-V map from Figure 2 at room temperature (Figure S2c).

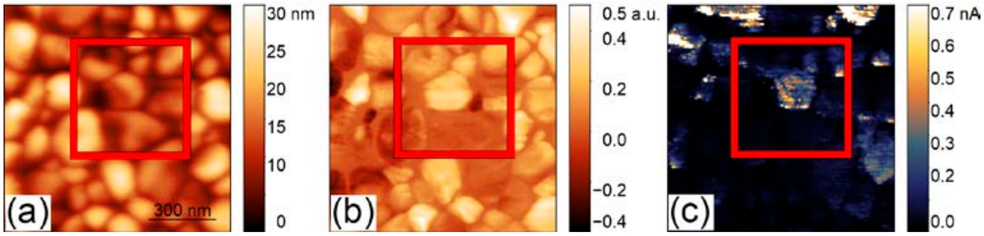

Figure S2. (a) Topography, (b) piezoresponse, and (c) CAFM of the region from Figure 2 in the main text of the manuscript. The red square denotes the region, where I-V map has been captured.

S3. Temperature dependencies of the area with the I-V curves of the different type

The temperature dependence of the areas with various types of I-V curves from Figure 3 in the main text of the manuscript is shown in Figure S3a. The gradual growth of the area with RS at the expense of other areas is visible. Figure S3b reveal a temperatures dependencies of the RS threshold voltage (green) and "cut-off" voltage of the registered current (orange)

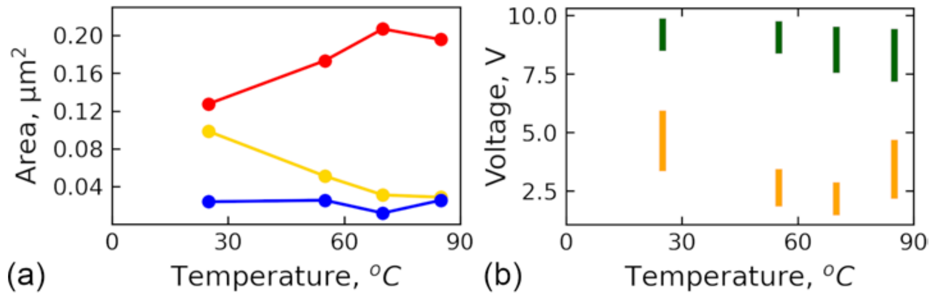

Figure S3. (a) Temperature dependencies of the area with the I-V curves of the different type with a color in correspondence to the Figure 2 from the main text of the paper. Red color – unipolar curves with RS, yellow color – unipolar curves without RS, and blue line – bipolar curves. (b) Temperatures dependencies of the RS threshold voltage (green) and "cut-off" voltage of the registered current (orange).

S4. X-ray photoelectron spectroscopy (XPS) of the BFO films

XPS was used to probe the defect state in BFO film, which is necessary to predict the type of conductivity [1–3]. Here, we skipped a detailed analysis of the survey spectrum and core levels of bismuth and oxygen and focus on the analysis of Fe $2p_{3/2}$ core level, which is meaningful to determine the type of defects in the material [4]. At first, the analysis was performed to discriminate between Fe^{2+} and Fe^{4+} states at the surface, which is associated with the formation of the oxygen vacancies compensated by the electrons or vacancies of bismuth or iron, associated with holes [3]. XPS spectra were collected from the BFO film, as-loaded to the vacuum chamber and after the shallow etching of the surface with an Ar ion beam (Figure S4a). First, we should note, that Fe^{3+} features at ~711 eV, ~719 eV, and ~724 eV were shifted to lower binding energies in comparison to a reference spectrum of Fe_2O_3 powder (Sigma Aldrich), but their positions were well coincident with those in the spectra of BFO films [5] and BLFO ceramics [4]. Around ~ 1.1 eV shift can be attributed to a net negative surface potential created by the negative built-in charge, similar to the case of bulk ceramics [4].

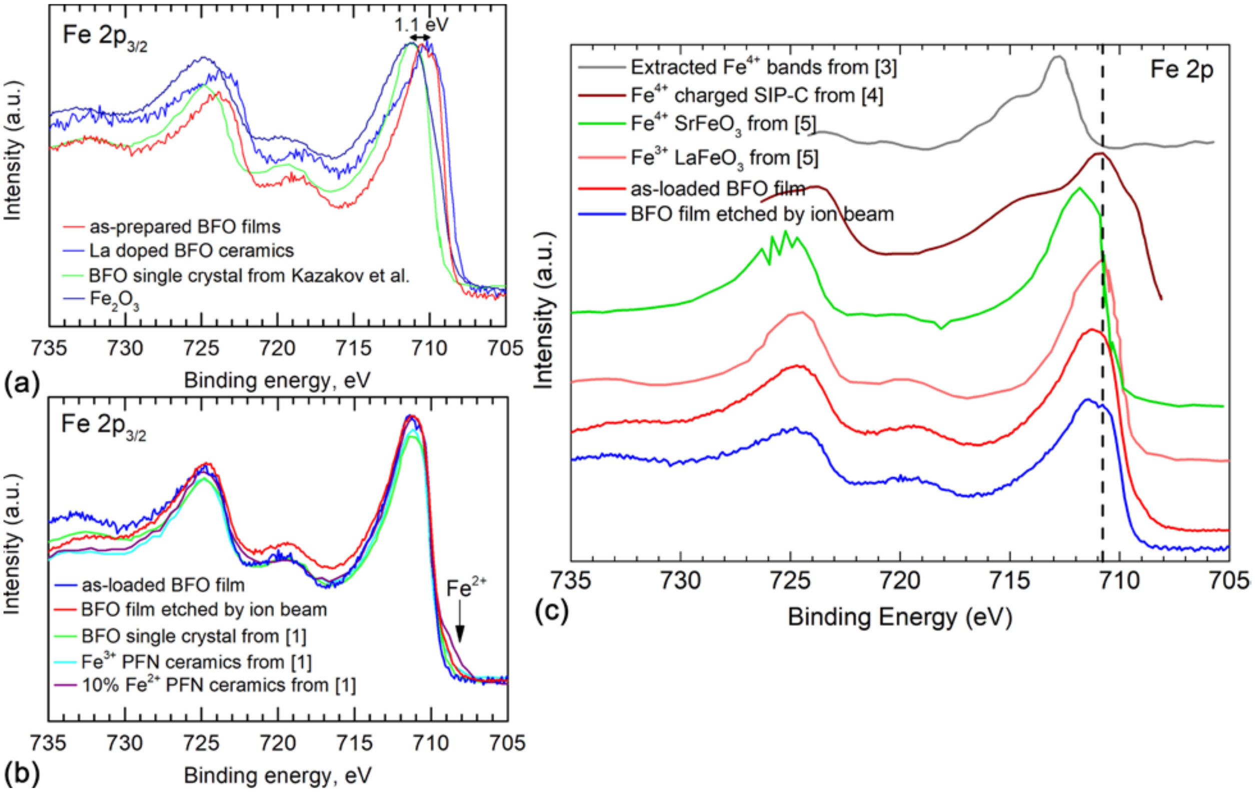

Figure S4. (a)-(c) Comparison of the measured Fe $2p_{3/2}$ XPS spectra of the as-loaded and etched by Ar ion beam BFO films with the reference literature data for the Fe_2O_3 powder, BFO single crystal, and modeled PFN ceramics spectra [6] and different literature reference data for the materials containing Fe^{4+} ions at the surface [6–9]: (a) before and (b), (c) after the shift of the spectra at 1.1 eV to higher binding energies. Fe^{2+} band at ~ 708 eV is indicated at (b).

The detailed inspection of XPS spectra after shift correction didn't reveal any features, indicating Fe^{2+} and Fe^{4+} in the spectrum (Figure S4b, S4c). According to Kozakov et al. [6] Fe^{2+}, the feature at 708 eV is expected (Figure S4c). Before ion etching, this feature is completely absent and the spectra are very similar to one for single-crystal BFO, while treatment by the Ar beam induces a small broadening at 708 eV, which is associated with the appearance of 3-5% of Fe^{2+}. The treatment by the ion beam stimulates the reduction of the material under radiation-stimulated local heating. Thus, Fe^{2+} state is expected to arise due to the formation of oxygen vacancies in the irradiated region of the film. As distinguished from BLFO ceramics [4], the surface also doesn't show much oxidization, and characteristic features of Fe^{4+}, widening of the ~712 eV and ~724 eV satellites, were not observed in the spectrum (Figure S4b, Figure S4c). The deconvolution could be performed well without including components of Fe^{4+} multiplet (Figure S4c).

Table S1. Atomic concentrations of the elements calculated from XPS spectra.

**Element**	**Atomic, %**	
	**As-loaded**	**Etched**
Bi	27.0	18.9
O	63.5	67
Fe	9.5	14.1
	**Ratio**	
Bi/Fe	2.8	1.3
O/Bi	2.4	3.3
O/Fe	6.7	4.8

Some conclusions can be given based on XPS element analysis (Table S1), which shows the concentration of bismuth to be almost 3 times higher than that of iron. Similar results were obtained for BLFO ceramics [4], and assume the migration of bismuth towards the surface. The off-stoichiometry is concentrated solely in the surface layer because X-ray diffraction didn't show the presence of the Bi-rich phase [10] and PFM revealed apparent piezoresponse contrast in most of the scanned areas. The significant off-stoichiometry at the surface can be associated with the volatility of the bismuth during crystallization. Despite the melting point of Bi_2O_3 being 817 °C [47], i.e. higher than the temperature of the synthesis, the surface off-stoichiometry can nevertheless be introduced due to the out-diffusion of bismuth. An excess of 7.5 mol% of bismuth nitrate allows to conserve stoichiometry in the material bulk but results in segregation of the bismuth at the surface, probably in the form of pure metal and bismuth oxide. Thus, we can expect the material to have bismuth vacancies in the subsurface layer. However, the formation of iron vacancies also cannot be excluded completely, keeping in mind that element analysis shows a lack of iron and formation energy for iron vacancies is closed to that for bismuth vacancies [2]. In both cases, the material conductivity type is likely p-type, but is closed to the intrinsic *n*=*p* semiconductor state, due to amount of Fe^{2+} and Fe^{4+} states in the film is very low (below XPS detection level).

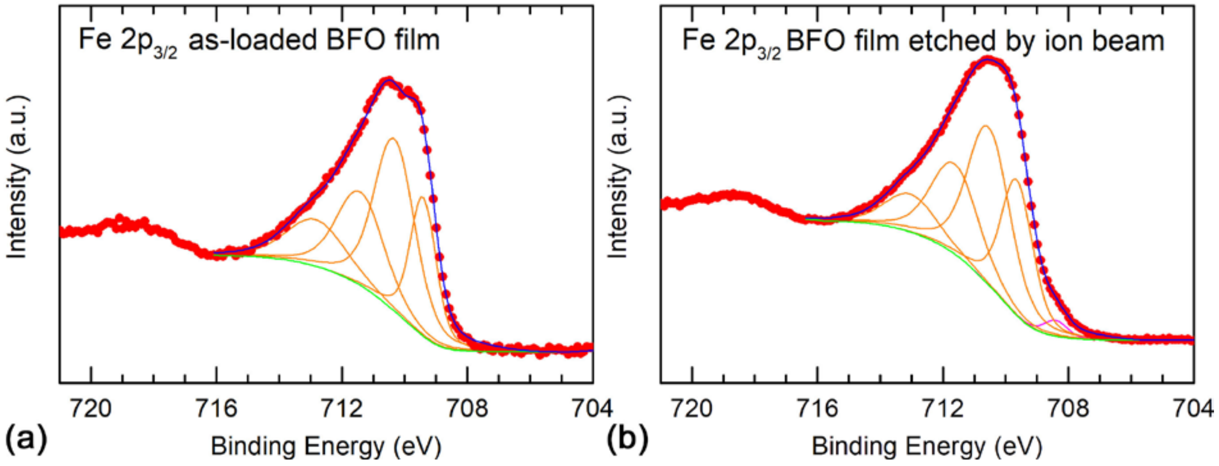

Figure S5. Deconvolution of Fe $2p_{3/2}$ band to Fe^{3+} (orange solid line) and Fe^{4+} (pink solid line) multiplet components for (a) as-loaded and (b) etched by Ar ion beam BFO films.

S5. Fitting of the I-V curves by equations of Schottky barrier

Non-hysteretic and hysteretic I-V curves from Figure 3e,i were fitted by equation (1) from the main text of the paper. These curves are presented in log(I) versus $U^{1/2}$ scale, in which linear behaviour represents fitting by equation 1 in the different regions of the curve (Figure S5). Equation (1) can be presented as: $I = Ae^{B\sqrt{U}}$, where coefficient B is equal to:

$$B = \frac{\sqrt{q^3}}{\sqrt{4\pi\varepsilon\varepsilon_0 d}(k_b T)}. \qquad \text{(S1)}$$

The value of the dielectric constant, ε, can be extracted from equation S1. In the different regions of the curves, dielectric constnt, ε, is ranged from 4 to 7, while it is expected to be around 30. This again confirms that the mechanism of the current transport is not a current through the Schottky barrier.

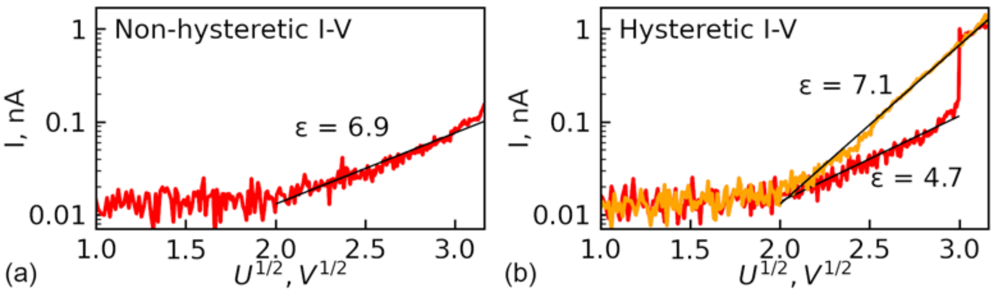

Figure S6. Fitting of the positive branch of the (a) non-hysteretic and (b) hysteretic I-V curves from Figure 3e,i by the equation (1) from the main text of the manuscript. Red and orange curves in (b) correspond to rice and fall of voltage.

S6. The distribution of the value of current "jump" during resistive switching

The values of the current "jump" during resistance switching were extracted for the red curves from Figure 2m-p and visualized as the pseudocolor images (Figure S6). It is clearly seen that the value of the "jumps" is increasing with temperature growth.

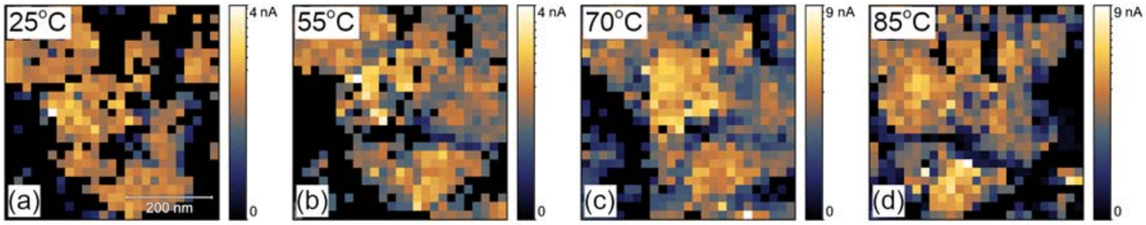

Figure S7. The maps of current "jump" during RS at (a) 25°C, (b) 55°C, (c) 70°C and (d) 85°C extracted from the analysis of the I-V curves.

S7. The analysis of the macroscopic I-V curve measured in capacitor geometry

Similar to our analysis of local I-V curves, the macroscopic curves from ref. [11] were analyzed (Figure S8). In region I before RS, the slope of the curve is almost linear, which indicates Ohmic contact. In region II the polarization reversal occurs, which leads to the release of electrons from the deep traps. As the measurements are macroscopic, the RS is visible not like a "jump", but represents a smoother curve, which is due to the distribution of the threshold voltages of the polarization reversal across the film and contribution to the electronic transport from the traps with the different energy. Simultaneously with the trap release, their refill by the electrons injected from the electrodes occurs, which is, in part, coincident with the consideration of the process in ref. [12]. After the complete screening of polarization, the power degree decreases to $m = 3.5$ and finally becomes equal to $m \approx 2$, which represents Mott-Gurney regime of the current flow in Region IV (LRS). Thus, the mechanism of the current transport in BFO is assumed to be a release of the electrons from the deep traps triggered by polarization reversal and aimed to screen polarization bound charge.

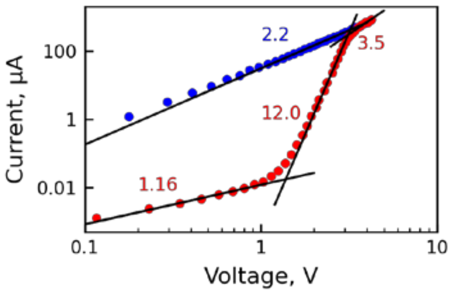

Figure S8. Analysis of the macroscopic I-V curve obtained for the epitaxial BFO film (Au/BFO(150 nm)/$SrRuO_3$ (20 nm)/$SrTiO_3$ (001) grown from a $Bi_{1.05}FeO_3$ target) from ref. [11] utilizing SCLC and trap release/refill model.

1. Masó, N.; West, A.R. Electrical Properties of Ca-Doped $BiFeO_3$ Ceramics: From p -Type Semiconduction to Oxide-Ion Conduction. *Chem. Mater.* **2012**, *24*, 2127–2132.
2. Zhang, Z.; Wu, P.; Chen, L.; Wang, J. Density functional theory plus U study of vacancy formations in bismuth ferrite. *Appl. Phys. Lett.* **2010**, *96*, 232906.
3. Wefring, E.T.; Einarsrud, M.-A.; Grande, T. Electrical conductivity and thermopower of $(1 - x)BiFeO_3 - xBi_{0.5}K_{0.5}TiO_3$ (x = 0.1, 0.2) ceramics near the ferroelectric to paraelectric phase transition. *Phys. Chem. Chem. Phys.* **2015**, *17*, 9420–9428.
4. Alikin, D.; Abramov, A.; Turygin, A.; Ievlev, A.; Pryakhina, V.; Karpinsky, D.; Hu, Q.; Jin, L.; Shur, V.; Tselev, A.; et al. Exploring Charged Defects in Ferroelectrics by the Switching Spectroscopy Piezoresponse Force Microscopy. *Small Methods* **2022**, *6*, 2101289.
5. Bein, N.S.; Machado, P.; Coll, M.; Chen, F.; Makarovic, M.; Rojac, T.; Klein, A. Electrochemical Reduction of Undoped and Cobalt-Doped $BiFeO_3$ Induced by Water Exposure: Quantitative Determination of Reduction Potentials and Defect Energy Levels Using Photoelectron Spectroscopy. *J. Phys. Chem. Lett.* **2019**, *10*, 7071–7076.
6. Kozakov, A.T.; Kochur, A.G.; Googlev, K.A.; Nikolsky, A.V.; Raevski, I.P.; Smotrakov, V.G.; Yeremkin, V.V. X-ray photoelectron study of the valence state of iron in iron-containing single-crystal ($BiFeO_3$, $PbFe_{1/2}Nb_{1/2}O_3$), and ceramic ($BaFe_{1/2}Nb_{1/2}O_3$) multiferroics. *J. Electron Spectros. Relat. Phenomena* **2011**, *184*, 16–23.
7. Šimša, Z.; Šimšová; Zemek, J.; Wigen, P.E.; Pardavi-Horvath, M. Search for Fe^{4+} in YIG : Ca Garnet Films. *Le J. Phys. Colloq.* **1988**, *49*, C8-975-C8-976.
8. Rajagopalan, R.; Chen, B.; Zhang, Z.; Wu, X.-L.; Du, Y.; Huang, Y.; Li, B.; Zong, Y.; Wang, J.; Nam, G.-H.; et al. Improved Reversibility of Fe^{3+}/Fe^{4+} Redox Couple in Sodium Super Ion Conductor Type $Na_3Fe_2(PO_4)_3$ for Sodium-Ion Batteries. *Adv. Mater.* **2017**, *29*, 1605694.
9. Wang, L.; Du, Y.; Sushko, P. V.; Bowden, M.E.; Stoerzinger, K.A.; Heald, S.M.; Scafetta, M.D.; Kaspar, T.C.; Chambers, S.A. Hole-induced electronic and optical transitions in $La_{1-x}Sr_xFeO_3$ epitaxial thin films Le. *Phys. Rev. Mater.* **2019**, *3*, 025401.
10. Abramov, A.; Alikin, D.; Sobol, A.; Myakishev, D.; Slabov, V.; Trusov, L.; Safina, V.; Turygin, A.; Vasiliev, A.; Shur, V.; et al. Chemical Solution Deposition of $BiFeO_3$ Films with Layer-by-Layer Control of the Coverage and Composition. *Coatings* **2020**, *10*, 438.
11. Chen, A.; Zhang, W.; Dedon, L.R.; Chen, D.; Khatkhatay, F.; MacManus-Driscoll, J.L.; Wang, H.; Yarotski, D.; Chen, J.; Gao, X.; et al. Couplings of Polarization with Interfacial Deep Trap and Schottky Interface Controlled Ferroelectric Memristive Switching. *Adv. Funct. Mater.* **2020**, *30*, 2000664.
12. Roy, P.; Kunwar, S.; Zhang, D.; Chen, D.; Corey, Z.; Rutherford, B.X.; Wang, H.; MacManus-Driscoll, J.L.; Jia, Q.; Chen, A. Role of Defects and Power Dissipation on Ferroelectric Memristive Switching. *Adv. Electron. Mater.* **2022**, *8*, 2101392.
